# Microbial pathways in colonic sulfur metabolism and links with health and disease

**DOI:** 10.3389/fphys.2012.00448

**Published:** 2012-11-28

**Authors:** Franck Carbonero, Ann C. Benefiel, Amir H. Alizadeh-Ghamsari, H. Rex Gaskins

**Affiliations:** ^1^Department of Animal Sciences, University of IllinoisUrbana, IL, USA; ^2^Division of Nutritional Sciences, University of IllinoisUrbana, IL, USA; ^3^Department of Pathobiology, University of IllinoisUrbana, IL, USA; ^4^Institute for Genomic Biology, University of IllinoisUrbana, IL, USA; ^5^University of Illinois Cancer Center, University of IllinoisUrbana, IL, USA

**Keywords:** sulfur, hydrogen sulfide, colonic microbiota, sulfate-reducing bacteria, inflammatory bowel disease, colorectal cancer, irritable bowel syndrome

## Abstract

Sulfur is both crucial to life and a potential threat to health. While colonic sulfur metabolism mediated by eukaryotic cells is relatively well studied, much less is known about sulfur metabolism within gastrointestinal microbes. Sulfated compounds in the colon are either of inorganic (e.g., sulfates, sulfites) or organic (e.g., dietary amino acids and host mucins) origin. The most extensively studied of the microbes involved in colonic sulfur metabolism are the sulfate-reducing bacteria (SRB), which are common colonic inhabitants. Many other microbial pathways are likely to shape colonic sulfur metabolism as well as the composition and availability of sulfated compounds, and these interactions need to be examined in more detail. Hydrogen sulfide is the sulfur derivative that has attracted the most attention in the context of colonic health, and the extent to which it is detrimental or beneficial remains in debate. Several lines of evidence point to SRB or exogenous hydrogen sulfide as potential players in the etiology of intestinal disorders, inflammatory bowel diseases (IBDs) and colorectal cancer in particular. Generation of hydrogen sulfide via pathways other than dissimilatory sulfate reduction may be as, or more, important than those involving the SRB. We suggest here that a novel axis of research is to assess the effects of hydrogen sulfide in shaping colonic microbiome structure. Clearly, in-depth characterization of the microbial pathways involved in colonic sulfur metabolism is necessary for a better understanding of its contribution to colonic disorders and development of therapeutic strategies.

## Introduction

The crucial role of the microbiome in digestive processes and intestinal health is now fully integrated in gastroenterology research (Gordon et al., 2007). The immense number of microbes resident in the small intestine and colon form complex communities and metabolic interactions, which impact the host beneficially or detrimentally. Accordingly, the intestinal microbiota plays a central role in digestive biochemistry and element cycling. Whereas abundant data are available regarding microbial carbon metabolism (carbohydrate or fiber degradation), less is known about microbial metabolism of other elements in the human intestine.

Historically, the sulfur cycle was one of the first to be well documented in environmental microbiology (Kertesz, 2000; Canfield and Farquhar, 2012). Chemolithoautotrophic and metabolic conversion of sulfur compounds were first described in the late 19th century by Beijerinck (Kelly et al., 1997) and are of central interest in the context of environmental pollution (Kellogg et al., 1972). Many sulfur compounds are also recognized for their toxicity and impact on human health (Ware et al., 1981). While there is no evidence for a microbial sulfur cycle *per se* in digestive systems, direct and indirect evidence points to a greater diversity and influence of microbial sulfur metabolism in the human intestine than previously recognized.

The sulfur present in the human body is provided exclusively by diet and either converted to sulfated compounds, assimilated by host cells or excreted. Sulfur is generally acquired in the human diet through protein (Stipanuk, 2004). It is noteworthy that one essential and another conditionally essential amino acid (methionine and cysteine, respectively) are sulfated, and thus, sulfur acquisition is crucial to humans. Cysteine is considered a conditionally essential amino acid as it can be synthesized from methionine via transsulfuration (Dominy and Stipanuk, 2004). As reviewed elsewhere, in addition to dietary inputs, host sulfur amino acids are actively recycled through a wide array of metabolic pathways (Stipanuk, 1986, 2004; Stipanuk and Dominy, 2006). Similar to the host, gut microbes require sulfur inputs and, because of their active metabolism and tremendous number, are likely to play a major role in the metabolism of luminal sulfur. Consequently, gut microbes are key players in determining the balance of beneficial to detrimental effects of sulfur-containing compounds (Table 1). This review summarizes microbial pathways influencing sulfur metabolism and their recognized and potential contributions to colonic health and disease.

**Table 1:** Microbial taxa involved in pathways of colonic sulfur metabolism^*^.

**Sulfur source**	**Sulfur-containing substrate**	**Microbial genus**	**References**
Inorganic	Sulfate (SO4^2−^)	*Desulfovibrio* spp. (*Desulfomonas* spp)	Gibson et al., 1988; Fite et al., 2004; Marchesi et al., 2009; Nava et al., 2012
		*Desulfobacter* spp.	Gibson et al., 1988; Nava et al., 2012
		*Desulfobulbus* spp.	Gibson et al., 1988; Nava et al., 2012
		*Desulfotomaculum* spp.	Gibson et al., 1988; Nava et al., 2012
	Sulfite (SO3^2−^)	*Bilophila wadsworthia*	Baron et al., 1989
		*Campylobacter jejuni*	Kelly and Myers, 2005
Organic	Cysteine	*Escherichia coli*	Metaxas and Delwiche, 1955; Shatalin et al., 2011
		*Staphylococcus aureus*	Shatalin et al., 2011
		*Salmonella thyphimurium*	Kredich et al., 1972
		*Mycobacterium tuberculosis*	Wheeler et al., 2005
		*Helicobacter pylori*	Kim et al., 2006
		*Leishmania major* (protozoa)	Nowicki et al., 2010
		*Prevotella intermedia*	Igarashi et al., 2009
		*Fusobacterium nucleatum*	Yoshida et al., 2010
		*Streptococcus anginosus*	Yoshida et al., 2011
		*Clostridium* spp.	Genomic cysteine desulfhydrase
		*Enterobacter* spp.	Genomic cysteine desulfhydrase
		*Klebsiella* spp.	Genomic cysteine desulfhydrase
		*Desulfovibrio* spp.	Genomic cysteine desulfhydrase
	Sulfomucin	*Prevotella* strain RS2	Roberton et al., 2000
		*Bacteroides fragilis*	Roberton et al., 2000
		*Helicobacter pylori*	Slomiany et al., 1992
		*Akkermansia* spp.	Genomic glycosulfatase
	Taurine	*Bilophila wadsworthia*	Laue et al., 1997
	Sulfated bile acids (rat model)	*Clostridium perfringens*	Huijghebaert and Eyssen, 1982; Huijghebaert et al., 1982; Robben et al., 1986
	Estrogen-3-sulfates and phenylsulfates	*Peptococcus niger*	Vaneldere et al., 1991

* Assimilatory sulfate reduction, widespread and present in virtually all microbes, has not been included.

## Intestinal sources of sulfur

Food sources of inorganic sulfate include commercial breads, dried fruits, vegetables, nuts, fermented beverages, and brassica vegetables (Florin et al., 1991). Diets supplemented with inorganic sulfate stimulate hydrogen sulfide (H_2_S) production within the colon (Christl et al., 1992; Lewis and Cochrane, 2007). Furthermore, *in vitro* incubation studies using human feces indicate that organic sulfur-containing compounds including cysteine, taurocholic acid, and mucin provide a more efficient source for sulfide production than inorganic sulfate (Florin, 1991; Levine et al., 1998), with meat being a particularly important source (Magee et al., 2000). In particular red meat, eggs, and milk contain elevated concentrations of cysteine. Concentrations of both free cysteine and methionine in colon are relatively low (Ahlman et al., 1993), indicating efficient metabolism by the microbiota and host epithelial cells. An additional source of colonic sulfur includes the sulfomucins. Mucins, consisting of a peptide backbone, are largely responsible for the viscous properties of the colonic mucus layer and can be broadly classified as sialomucins or sulfomucins based on the presence of terminal sialic acid or sulfate groups, respectively, on the oligosaccharide chain (Verdugo, 1990; Jass and Roberton, 1994; Croix et al., 2011).

Many other trace sulfated compounds are provided by dietary elements. In particular, fermented foodstuffs contain a wide array of volatile sulfur compounds (Landaud et al., 2008). Microbial and host cell metabolism also produce a large variety of simple to complex sulfated compounds available for further microbial utilization or degradation.

## Microbial pathways involved in colonic sulfur metabolism

### Assimilatory sulfate reduction

**Figure 1:**
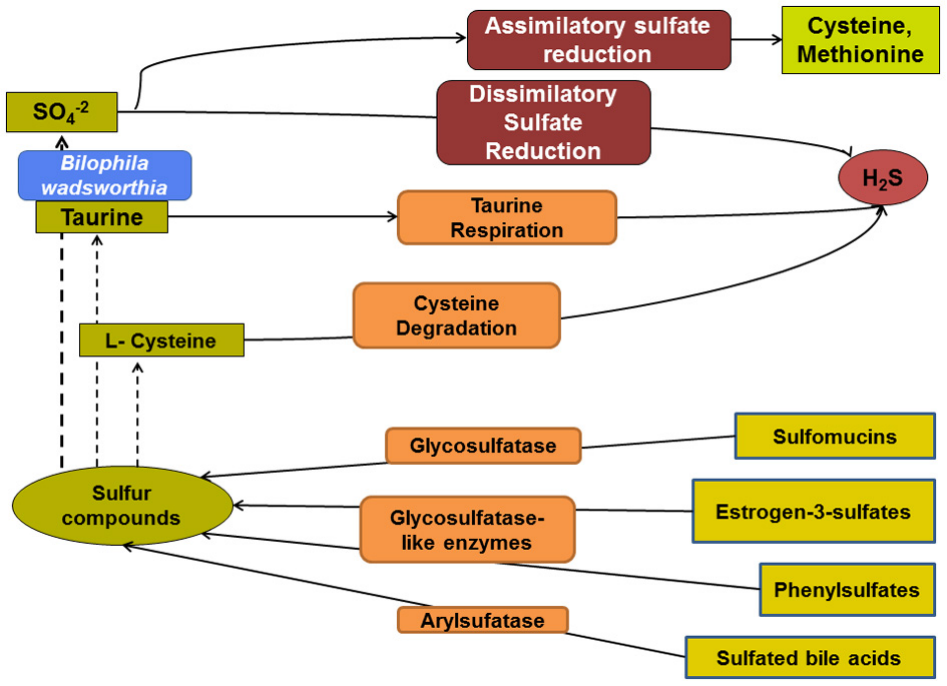
**Overview of the microbial pathways involved in colonic sulfur metabolism.** The sulfate remaining from assimilatory sulfate reduction is available for sulfate-reducing bacteria and, thus, H_2_S production. Taurine and cysteine are additional potentially important substrates for microbial production of H_2_S. Various microbial metabolic pathways influence the composition and relative abundance of organic sulfur compounds.

Among microbes, assimilatory sulfate reduction is the most widespread and essential biochemical process linked to sulfur and provides a mechanism by which microorganisms can reduce sulfate to elemental sulfur in order to satisfy physiological requirements (Peck, 1961). This process is mediated by phosphoadenosine-5′-phosphosulfate (PAPS) reductases (Figure 1), which are widely distributed among microbes and other living organisms except animals (for example 2924 hits for annotated nucleotide sequences and 10,402 for protein sequences were found in the NCBI database). Assimilatory sulfate reduction can also be performed by other enzymatic pathways (Vaneldere et al., 1991; Seitz and Leadbetter, 1995) and likely represents a crucial step in sulfur cycling as it allows for significant disposal of sulfate and conversion to necessary organic sulfated compounds.

### Dissimilatory sulfate reduction

**Figure 2:**
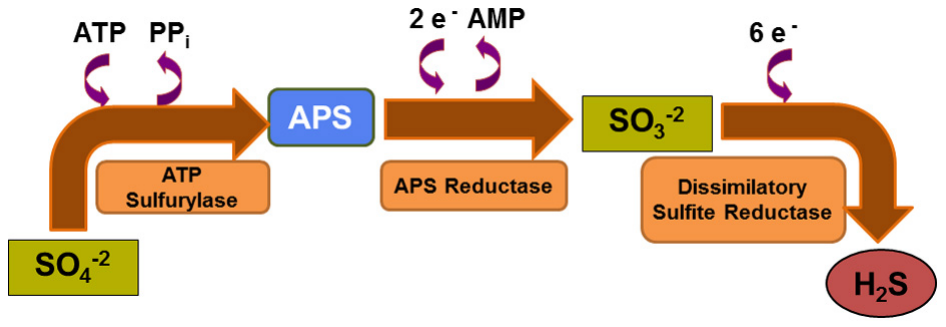
**Dissimilatory sulfate reduction pathway.** Microbial sulfate reduction relies on sequential catalytic reactions in which reduction of sulfate is coupled with oxidation of H_2_ or simple organic molecules. This anaerobic respiration pathway is less favorable thermodynamically than aerobic respiration.

While the process of assimilatory sulfate reduction is widespread among microbes, only restricted microbial groups are capable of dissimilatory sulfate reduction (Figure 2). The sulfate-reducing bacteria (SRB) are notable as the only microbes in intestinal ecosystems that rely on inorganic sulfate for conservation of energy. This sulfate respiration pathway has been conserved in five bacterial and two archaeal phyla (Wagner et al., 1998). Most of the SRB belong to the Deltaproteobacteria with more than 25 genera, followed by the Gram-positive SRB within the Clostridia (*Desulfotomaculum, Desulfosporosinus*, and *Desulfosporomusa* genera). The SRB use sulfate as a terminal electron acceptor for respiration, with the concomitant production of H_2_S (Peck, 1961). In the colon, sulfate reduction is generally associated with dihydrogen oxidation (Carbonero et al., 2012), but the electrons may also be provided from the oxidation of organic compounds, such as lactate (Flint et al., 2009). SRB are ubiquitously present in the human intestinal mucosa (Nava et al., 2012) and have been enumerated by cultivation-dependent methods from human stool in numbers ranging from 10^3^ to 10^11^/g (Leclerc et al., 1980; Gibson et al., 1988). In a culture-based study by Gibson et al., the principal SRB were lactate- and hydrogen-utilizing *Desulfovibrio* spp. (64–81%), acetate-utilizing *Desulfobacter* spp. (9–16%), propionate- and hydrogen-utilizing *Desulfobulbus* spp. (5–8%), lactate-utilizing *Desulfomonas* spp (reclassified within the genus *Desulfovibrio*) (3–10%), and acetate- and butyrate-utilizing *Desulfotomaculum* spp. (2%). However, these observations are based on cultivation methods, which underestimate true bacterial diversity. More recently molecular-based techniques have been applied successfully to describing SRB diversity in various environments. SRB have thus far been detected and quantified from stool and colonic mucosa samples (Zinkevich and Beech, 2000; Fite et al., 2004; Nava et al., 2012). The genus *Desulfovibrio* has generally been found in higher numbers (Fite et al., 2004; Marchesi et al., 2009), with lower abundances of *Desulfobacter, Desulfobulbus*, and *Desulfotomaculum* (Nava et al., 2012). The biochemistry of dissimilatory sulfate reduction has been investigated most extensively within species of *Desulfovibrio*. In the environment, SRB are able to utilize a wide range of substrates as electron donors and acceptors and can even adopt non-sulfidogenic lifestyles (Plugge et al., 2011). For example *Desulfobulbus* spp. can ferment organic matter under certain conditions (Kuever et al., 2005). It is not known if colonic SRB possess the ability to switch from sulfidogenic to non-sulfidogenic lifestyles in the colon, but an ability to adapt to varying sulfate levels would confer a competitive advantage.

### Cysteine degradation

**Figure 3:**
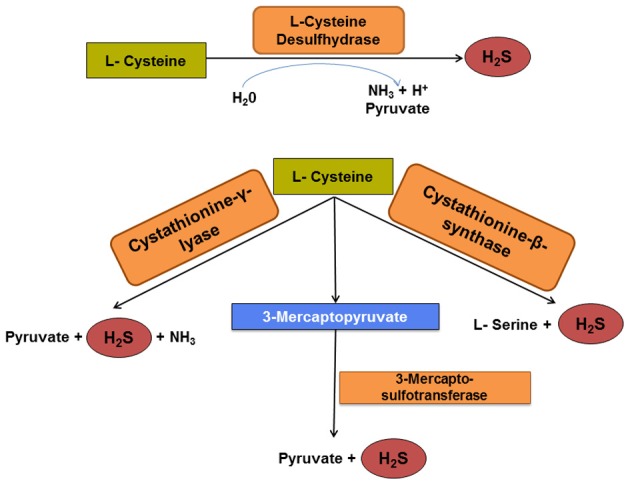
**Pathways of cysteine degradation to H_2_S.** Cysteine desulfhydrase is a key enzyme for initial microbial cysteine fermentation and pyruvate production. However, recent evidence indicates that three other enzymes orthologous to eukaryotic enzymes may catalyze similar reactions. The generation of H_2_S via these four enzymatic pathways may surpass that by dissimilatory sulfate reduction, especially in hosts consuming a protein-rich diet.

Cysteine degradation to H_2_S mediated by L-cysteine desulfhydrase (Figure 3) was described in the 1950s in the *Escherichia coli* model, a well-known inhabitant of the human gut (Metaxas and Delwiche, 1955). Since that time, L- or D-cysteine desulfhydrase have been described in diverse taxa (Kumagai et al., 1975) including a few intestinal pathogens such as *Salmonella thyphimurium* (Kredich et al., 1972), *Mycobacterium tuberculosis* (Wheeler et al., 2005), *H. pylori* (Kim et al., 2006), and the protozoan *Leishmania major* (Nowicki et al., 2010). L- or D-cysteine desulfhydrase have also been studied in various oral microorganisms as a source of malodor and abscess formation (Igarashi et al., 2009; Yoshida et al., 2010, 2011). Those genera found in the oral cavity, namely *Streptococcus, Prevotella* and *Fusobacterium*, are also common inhabitants of the human colon. A search in the GenBank and EBI databases revealed that cysteine desulfhydrase-encoding genes have been annotated in several common colonic genera such as *Clostridium, Enterobacter, Klebsiella, Streptococcus*, and, surprisingly, the SRB genus *Desulfovibrio*. Although desulfhydrase activity has not been quantified in intestinal ecosystems and the prevalence of the gene among intestinal taxa remains undefined, it appears likely that desulfhydrase-harboring microbes are important producers of H_2_S in the human colon.

Other microbial enzymes possess cysteine desulfhydrase activity, and, more generally, the ability to convert cysteine or cysteine analogs is widespread. Cysteine desulfidases, that carry out reactions similar to other desulfhydrases, have been described in environmental archaea (Tchong et al., 2005). Beta C-S lyases or cystathionases mediates the cleavage of cystathionine to homocysteine in *E. coli* (Zdych et al., 1995) and *Lactobacillus* spp. (De Angelis et al., 2002; Irmler et al., 2008). Another example is the SRB *Desulfovibrio desulfuricans*, which is able to obtain sulfate from cysteine degradation (Forsberg, 1980). Conversely, the prevalent O-acetyl-L-serine sulfhydrylase (OASS) and homocysteine synthase mediate the microbial synthesis of cysteine from O-acetyl-L-serine and sulfide in bacteria and archaea (Borup and Ferry, 2000; Tai and Cook, 2001).

A recent paper provided evidence that a high proportion of bacterial genomes harbor orthologs of mammalian cystathionine β-synthase (CBS), cystathionine γ-lyase (CSE), and 3-mercaptopyruvate sulfurtransferase (3MST), three enzymes involved in H_2_S production (Shatalin et al., 2011). The authors confirmed that pathogenic strains, including *E. coli* and *Staphylococcus aureus*, converted cysteine to H_2_S through activities of one of those enzymes. Thus, it may be hypothesized that many gut microbes are capable of cysteine conversion to H_2_S. Further, the authors demonstrated that sulfide production reduces oxidative stress, thereby increasing antibiotic resistance potential (Shatalin et al., 2011). Indeed, those microbial metabolic pathways are very poorly documented in gut commensals, and characterization of these enzymes and associated genes will be crucial to understanding if microbial-generated sulfide plays a role in shaping the colonic ecosystem.

### Taurine and sulfite respiration

**Figure 4:**
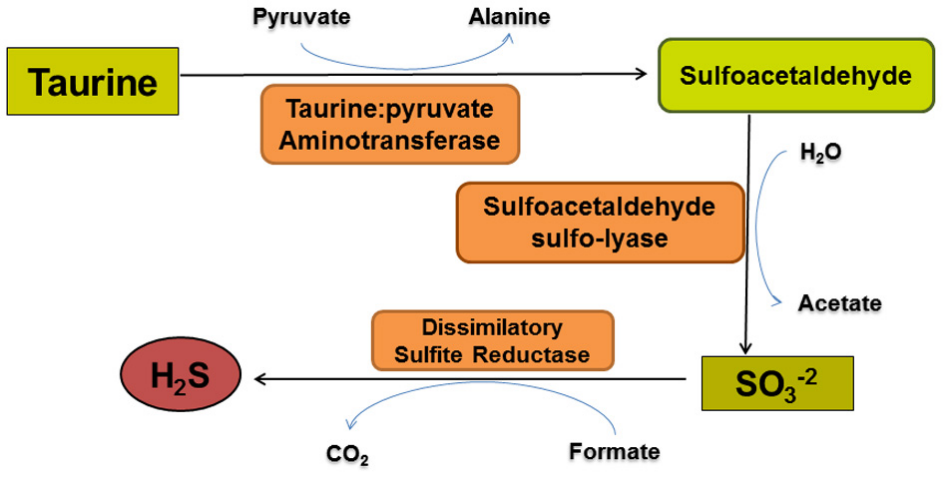
**The taurine degradation pathway of *Bilophila wadsworthia*.** *Bilophila wadsworthia* is the only known intestinal microbe that uses taurine as an electron acceptor for anaerobic respiration. The first two enzymatic reactions result in sulfite production. Sulfite is subsequently converted to H_2_S by a dissimilatory sulfite reductase that differs structurally from those used by sulfate-reducing bacteria.

Only a few genera are able to gain energy through sulfite respiration rather than direct sulfate respiration (Pukall et al., 1999; Soulimane et al., 2011). Among those, the only intestinal resident characterized to date is *Bilophila wadsworthia*, a close relative of *Desulfovibrio* spp. (Baron et al., 1989). This unique bacterial species has attracted much attention because of its links with various disease symptoms (Finegold et al., 1992; Arzese et al., 1997; Aucher et al., 1998; Nakayama et al., 2000; Devkota et al., 2012). It reduces sulfite after an initial degradation of taurine and harbors a different dissimilatory sulfite reductase than other SRB (Laue et al., 2001) (Figure 4). A sulfite oxidation metabolic pathway has also been demonstrated in the food-borne human pathogen *Campylobacter jejuni* (Kelly and Myers, 2005); however, the existence of similar pathways in intestinal commensal microbes has not been described.

Taurine, a sulfated compound secreted by the eukaryotic host that is crucial for chelating bile acids in addition to contributing to osmoregulation, cardiac function, and retinal development is degraded by *B. wadsworthia* using a taurine:pyruvate aminotransferase to obtain sulfite (Laue et al., 1997; Laue and Cook, 2000) (Figure 4). An intriguing link between dietary fat and the utilization of taurochloric acid as a source of taurine for *B. wadsworthia* is suggested by two recent studies (Swann et al., 2011; Devkota et al., 2012). Compared to mice fed a low fat or high polyunsaturated fat diet, mice fed a high milk fat diet demonstrated an overrepresentation of *B. wadsworthia*, which was determined to be mediated by taurine-conjugated bile acids (Devkota et al., 2012). Furthermore, milk fat was essential for colonization by *B. wadsworthia* in the germ-free mouse colon. Swann et al. (2011) demonstrated that the lack of an established microbiota results in a dominance of taurine-conjugated bile acids over unconjugated or glycine-conjugated bile acids, suggesting that bile acids, whose composition is modulated by the gut microbiota, may serve as signaling molecules in liver and other tissues (Swann et al., 2011). Although taurine has also been demonstrated to be a substrate for SRB in marine microbial mats (Visscher et al., 1999), it is not known if colonic SRB are capable of taurine utilization.

### Arylsulfatases

Bile acids can be sulfated by the action of host sulfotransferases, which allow their subsequent detoxification and disposal (Alnouti, 2009). It was demonstrated in a rat model that the opposite conversion, bile acid desulfation, can be mediated by microbial enzymes with arylsulfatase activity (Robben et al., 1988). Arylsulfatases were purified from *Clostridium perfringens* (Huijghebaert and Eyssen, 1982; Huijghebaert et al., 1982; Robben et al., 1986) inhabiting the rat intestine and are also widespread in several abundant colon genera. Little arylsulfatase activity was reported in human stool (McBain and Macfarlane, 1998), which does not preclude a more extensive activity of microbial arylsulfatase in the colonic ecosystem where significant concentrations of sulfated bile acids are found (Palmer, 1967; Hofmann et al., 2008).

### Glycosulfatases

Some colonic microbes have evolved to degrade colonic mucins (Roberton and Stanley, 1982; Derrien et al., 2004, 2008). In particular, microbial glycosulfatases catalyze the release of sulfate from sulfomucins (Corfield et al., 1992; Roberton and Wright, 1997). Glycosulfatase enzymes have been described primarily in the intestinal resident *Prevotella* strain RS2 and *Bacteroides fragilis* (Roberton et al., 2000), but also in the stomach resident *Helicobacter pylori* (Slomiany et al., 1992). A suite of glycosulfatase-like enzymes were also described in the intestinal *Peptococcus niger*, using less abundant substrates such as estrogen-3-sulfates and phenylsulfates (Vaneldere et al., 1991). Relative abundance and activity of glycosulfatase-harboring bacteria may greatly influence colonic sulfur metabolism; however, little is known about prevalence, abundance, and activity of bacterial glycosulfatases. A search in the GenBank and EBI databases confirmed that glycosulfatases encoding genes have been annotated in important colonic genera such as *Prevotella, Bacteroides* (Arumugam et al., 2011), and *Akkermansia* (Belzer and De Vos, 2012).

## Links with colonic health and disease

### Hydrogen sulfide

#### Toxicity

Hydrogen sulfide is the most suspect sulfated compound in the etiology of colonic disorders and inflammation (Nakamura et al., 2010; Danese and Fava, 2011; Medani et al., 2011). In addition, it is among the most hazardous gases in industrial applications, exhibiting toxicity to different organs at low concentrations, with increasing concentrations being fatal (Guidotti, 1996). There is ample evidence that H_2_S at physiological concentrations is genotoxic and induces DNA damage as well as inflammatory responses (Attene-Ramos et al., 2006, 2007, 2010). Exogenous hydrogen sulfide is highly toxic to colonocytes and impairs their metabolic function, especially butyrate oxidation (Roediger et al., 1993; Babidge et al., 1998). In the human colon, sulfide exists largely in the volatile, highly toxic undissociated form (H_2_S), which is quickly absorbed by the mucosa or passes as flatus (Suarez et al., 1997). Over 90% of sulfate disappears during passage through the colon of individuals lacking a sulfate-reducing microbiota, indicating that additional colonic processes compete for sulfate (Strocchi et al., 1993).

#### Detoxification

Hydrogen sulfide is oxidized in many tissues including colonic mucosa (Bartholomew et al., 1980; Furne et al., 2001), which presumably is exposed frequently to bacterial-derived sulfide, given the persistent colonization of the human colonic mucosa by SRB (Nava et al., 2012). The first step in the sulfide oxidation pathway is catalyzed by sulfide quinone reductase (SQR), a mitochondrial flavoprotein that oxidizes H_2_S to protein-bound persulfide. Subsequently, a sulfur dioxygenase and a sulfur transferase are thought to sequentially convert SQR-bound persulfide into sulfite and thiosulfate, respectively (Wilson et al., 2008; Kabil and Banerjee, 2010). Given that most of the colonic disorders for which a potential pathogenic role for sulfide exists are effectively gene-environment disorders, it is straight-forward to envision how common polymorphisms in host genes encoding components of the sulfide oxidation pathway might underlie ineffective epithelial sulfide detoxification and thereby predispose certain individuals to chronic sulfide-generated inflammation or genotoxicity.

#### H_2_S as an endogenous signaling molecule

Despite its potential inflammatory, toxic and genotoxic properties, there is also evidence that human cells may be able to use H_2_S as a inorganic substrate for mitochondrial energization (Bouillaud et al., 2007). It is postulated that H_2_S may be an important endogenous signaling molecule (Abe and Kimura, 1996; Distrutti et al., 2006). In addition, H_2_S has been suggested to be an effective drug against various GI diseases in a rodent model (Wallace et al., 2007, 2009; Wallace, 2010). However, the potential therapeutic properties of exogenous H_2_S still remain controversial (Bouillaud and Blachier, 2011; Olson, 2011). Intriguingly, a recent report demonstrated that inhibition of H_2_S synthesis in healthy rats resulted in mucosal injury and inflammation in the small intestine and colon, while intracolonic administration of H_2_S donors significantly reduced the severity of trinitrobenzene sulfonic acid-induced colitis (Wallace et al., 2009). These data indicate that the outright assumption that colonic H_2_S is only deleterious may be challenged and justify additional study of both bacterial and endogenous sources of H_2_S in the human colon.

#### H_2_S as an architect of microbiome structure

In the context of culture-based microbiology, sulfide in different forms has been used as a strong reducing agent, allowing maintenance of anoxia in media. However, sulfide concentrations must be kept low to avoid toxicity in microbial cells. For example, in microcosms simulating anaerobic digesters, lactose fermentation and methanogenesis were inhibited by sulfide in a dose-dependent manner. Interestingly, it was also observed that SRB activities were inhibited by increased sulfide concentrations (Hilton and Oleszkiewicz, 1988). In surface sediments of sandy intertidal flats, high sulfide concentrations may reduce functional diversity (Freitag et al., 2003). Finally, sulfide production was shown to provide cytoprotection from oxidative stress (Shatalin et al., 2011). As a start, *in vitro* studies with representative colonic strains are needed to better understand the potential for sulfide as well as sulfated compounds to influence the structure of colonic ecosystem.

### Inflammatory bowel disease

The two major forms of inflammatory bowel disease (IBD), Crohn's disease (CD), and ulcerative colitis (UC), afflict 0.1–0.5% of individuals in Western countries (Podolsky, 2002; Hanauer, 2006). It is commonly accepted that IBD results from multifactorial interactions among genetic and environmental factors that lead to a dysregulation of the innate immune response to the intestinal microbiota in genetically predisposed individuals (Podolsky, 2002).

Substantial evidence exists for a potential pathogenic role of H_2_S in IBD, particularly in UC (Pitcher and Cummings, 1996). Healthy colonic epithelial cells depend on the availability of short-chain fatty acids such as butyrate for nutrition. Butyrate is produced during colonic fermentation and oxidized by colonocytes via the enzyme acyl-CoA dehydrogenase. Because this enzyme is inhibited by H_2_S, oxidation of butyrate is impaired by H_2_S (Babidge et al., 1998). Patients with UC were shown to have reduced breath excretion of CO_2_ after administration of butyrate intrarectally, reflecting reduced colonic butyrate oxidation (Den Hond et al., 1998). UC patients with reduced colonic butyrate oxidation also exhibit increased intestinal permeability (Den Hond et al., 1998). Indeed, as ion absorption, mucin synthesis, membrane lipid synthesis, and detoxification processes in colonocytes depend on the oxidation of butyrate (Roediger et al., 1997), decreases of butyrate oxidation would be expected to compromise epithelial barrier function (Ramakrishna et al., 1991).

Patients with UC have been shown to consume more protein and, thereby, more sulfur amino acids than control subjects (Tragnone et al., 1995). Removing foods such as milk, cheese, and eggs improved symptoms in UC patients (Truelove, 1961). Further, the numbers of SRB and rate of sulfidogenesis were reported greater in UC patients than control cases (Gibson et al., 1991; Pitcher et al., 2000). In a study comparing patients with IBD to healthy individuals or patients with other gastrointestinal symptoms, the prevalence of *Desulfovibrio piger* detected via PCR was significantly higher in the IBD patients (Loubinoux et al., 2002). Levine et al. (1998) found that production of H_2_S from feces of patients with UC was 3–4 times greater than from feces of healthy controls. However, this difference in H_2_S production may not have been due to colonization by a greater number of SRB, as qPCR did not show that patients with active UC harbored more SRB in stool or rectal mucosa than healthy controls (Fite et al., 2004). A common treatment regimen for patients with UC may contribute to conflicting results regarding the density of SRB populations in UC patients. Specifically, 5-aminosalicylic acid (5-ASA), an anti-inflammatory medication commonly prescribed for UC, also inhibits SRB growth and production of H_2_S (Pitcher et al., 2000; Edmond et al., 2003). For example, stool sulfide concentrations between patients with UC and non-colitic controls did not differ when the use of salicylates in colitic patients was not controlled (Moore et al., 1998); in those patients with UC who were not administered 5-ASA, fecal sulfide concentrations were significantly greater (Pitcher et al., 2000).

Additional evidence for the role of H_2_S in UC is the observation that SRB were found in surgically constructed ileo-anal pouches of UC patients but not in pouches of patients with familial adenomatous polyposis (FAP). Furthermore, H_2_S production in UC pouches was 10 times greater than that in FAP pouches (Duffy et al., 2002). Finally, in a later study, the severity of pouchitis was positively correlated with fecal concentrations of H_2_S (Ohge et al., 2005), possibly reflecting a pathogenic role for this gas. Coffey et al. (2009) proposed that the surgical creation of ileo-anal pouches in UC patients might lead to colonic metaplasia, which, in turn, could result in increased production of sulfomucin and, thus, higher colonization by SRB. The adverse consequences of such colonization would be greater exposure to potentially proinflammatory concentrations of H_2_S (Coffey et al., 2009).

Increased activity of mucin sulfatase, an enzyme belonging to the glycosulfatase group, was observed in patients with active UC but not CD (Tsai et al., 1995). In most patients, fluctuations in fecal sulfatase activity corresponded with severity of clinical disease, suggesting that the increased fecal sulfatase activity contributed to perpetuation of the disease. In this scenario, individuals genetically predisposed to a high SRB carriage rate who then experience increased sulfatase activity might be at increased risk of UC due to the increased availability of endogenous sulfate for SRB sulfide production. Similarly, diets high in exogenous sources of sulfate would represent the greatest risk for those genetically predisposed to a higher abundance of SRB.

Recently, a culture-dependent study demonstrated that mucosal biopsies from IBD patients were more often colonized by *Fusobacterium* spp. than those from matched healthy controls (Strauss et al., 2011). It was further demonstrated that *Fusobacterium* isolates from IBD patients trigger inflammatory pathways in colonic cells (Dharmani et al., 2011). *Fusobacterium* spp. produce H_2_S through cysteine desulfhydrase activity. It also appears possible that direct consumption of cysteine can reduce in some instances sulfate availability for SRB, thereby possibly explaining the conflicting reports of SRB association with IBD. The recent demonstration of selection for *B. wadsworthia* in the gut microbiota of mice fed a high-saturated fat diet that was associated with higher occurrence and severity of colitis compared to those fed high-unsaturated fat (Devkota et al., 2012) provides a potential mechanistic model for the development of IBD in susceptible individuals. Notably, this effect was mediated by an increase of specific bile acids, particularly taurocholic acid, a potential source of taurine for *B. wadsworthia*.

### Colorectal cancer

Colorectal cancer is the third most frequent cancer worldwide and the fourth most common cause of cancer death, with responsibility for 4,92,000 deaths annually (Weitz et al., 2005; Ferlay et al., 2010). Genetic and environmental factors play a significant role in the development of colorectal cancer (Kinzler and Vogelstein, 1996; Rhodes and Campbell, 2002; de la Chapelle, 2004). Although etiologically divided into sporadic (90% of the cases), hereditary (5–10%), and IBD-associated (2%), all colorectal cancers show multistep development with several mutations (Kinzler and Vogelstein, 1996; Rhodes and Campbell, 2002; de la Chapelle, 2004). Doll and Peto (1981) estimated that over 90% of gastrointestinal cancers are determined by environmental factors such as diet. It has been suggested that environmental cancer risk is determined by the interaction between diet and colonic microbial metabolism (O'Keefe et al., 2007). Particularly, there is strong epidemiologic and experimental evidence that diets with high animal fat and protein (meat) are associated with increased risk of colorectal cancer (Willett et al., 1990; Sandhu et al., 2001; Norat et al., 2002). As discussed earlier, meat contains relatively high dietary sulfur, which can promote microbial sulfate reduction in the colon.

Kanazawa and colleagues (1996) demonstrated that H_2_S concentrations were significantly greater in patients who had previously undergone surgery for sigmoid colon cancer and who later developed new epithelial neoplasia of the colon, compared to individuals of similar age with a healthy colon. The ability of the colon to detoxify H_2_S is also reduced in patients with colon cancer (Ramasamy et al., 2006). The association of H_2_S with colon cancer is further supported by the finding that H_2_S induces colonic mucosal hyperproliferation, with this effect reversed by butyrate (Christl et al., 1996). The mucosal hyperproliferation effect of H_2_S may be mediated by mitogen-activated protein kinase (MAPK)-mediated proliferation (Deplancke and Gaskins, 2003). Hydrogen sulfide is also a potent genotoxin that induces direct radical-associated DNA damage (Attene-Ramos et al., 2006, 2007). Colon cancer in UC and, perhaps, sporadic colon cancer in general may reflect genomic instability resulting from exposure to H_2_S (Attene-Ramos et al., 2007). As the number of SRB was reported to be not significantly different (Balamurugan et al., 2008) or reduced in colorectal cancer patients when compared to healthy controls (Scanlan et al., 2009), impaired detoxification of H_2_S may be critical to the role of this compound in colon cancer.

Increased detection of *Fusobacterium* spp. in CRC tumors has been reported in two independent studies (Kostic et al., 2011; Castellarin et al., 2012). As described previously, *Fusobacterium* spp. produce H_2_S through cysteine desulfhydrase activity. As H_2_S has been demonstrated to be genotoxic, it can be hypothesized that it may be one causative metabolite in the etiology of CRC. It also appears possible that direct bacterial consumption of cysteine would limit sulfate availability for SRB, thereby possibly explaining the conflicting reports of SRB association with CRC.

### Irritable bowel syndrome

Irritable bowel syndrome (IBS) is a functional bowel disorder characterized by chronic abdominal pain, bloating, and abnormal bowel habits (Mayer, 2008). Diarrhea or constipation may predominate or these symptoms may alternate. Microbial dysbioses have been described, and as microbial gases excreted in breath are higher in IBS patients, abnormal microbial pathways are a possible cause (King et al., 1998).

Consistent with this possibility, it was demonstrated recently that exogenous H_2_S (NaHS) inhibits *in vitro* motor patterns in the human, rat and mouse colon and jejunum mainly through an action on multiple potassium channels (Gallego et al., 2008). Exogenous H_2_S (NaHS) inhibited nociception induced by colorectal distension in both healthy and post-colitic rats (Distrutti et al., 2006) but, conversely, triggered visceral nociceptive behavior when administered intracolonically in the mouse (Matsunami et al., 2009). Recently, a functional dysbiosis was described in constipation-predominant IBS patients with, in particular, a significant increase of SRB and fecal sulfide, indicating a potential role for sulfide in the development of IBS symptoms (Chassard et al., 2012).

## Conclusions

Microbial sulfur metabolism in the human colon is likely more extensive than has been previously recognized. For example, H_2_S, the sulfated compound with the highest potential influence on digestive health, can be generated from cysteine degradation as well as via dissimilatory sulfate reduction. The extent to which organic sulfur metabolism is operative in the human colon, how diet influences relative activities of the enzymatic pathways involved, and which organisms carry out these processes are poorly understood. In addition, the importance of the microbiota and the metabolism of sulfated bile acids are now established, and further work is needed to understand how dietary fat intake influences these pathways. With clear evidence that the human colonic mucosa is persistently colonized by SRB, the beneficial versus toxic effects of H_2_S need to be delineated. One possibility is that the mucosal microbiome is shaped in part through the differential susceptibility of mutualistic microbes to sulfide toxicity. Clearly, extensive work is needed to understand the primary pathways for the production of H_2_S and how their activities may influence both host and microbial components of the colonic ecosystem. This new body of knowledge may identify relatively innocuous approaches for the prevention or treatment of chronic colonic disorders.

### Conflict of interest statement

The authors declare that the research was conducted in the absence of any commercial or financial relationships that could be construed as a potential conflict of interest.
